# A systematic study on the synergistic effects of MWCNTs and core–shell particles on the physicomechanical properties of epoxy resin

**DOI:** 10.1038/s41598-021-00333-3

**Published:** 2021-10-21

**Authors:** Ali Gharieh, Mir Saeed Seyed Dorraji

**Affiliations:** 1 Department of Polymer Chemistry, Faculty of Chemistry, University of Isfahan, Isfahan, 81746-73441 Iran; 2 Applied Chemistry Research Laboratory, Department of Chemistry, Faculty of Science, University of Zanjan, Zanjan, Iran

**Keywords:** Chemistry, Materials science, Nanoscience and technology

## Abstract

Here, core–shell impact modifier particles (CSIMPs) and multiwalled carbon nanotubes (MWCNs) were used as reinforcing agents for improving the toughness and tensile properties of epoxy resin. For this purpose, emulsion polymerization technique was exploited to fabricate poly(butyl acrylate-allyl methacrylate) core-poly(methyl methacrylate-glycidyl methacrylate) shell impact modifier particles with an average particle size of 407 nm. It was revealed that using a combination of the prepared CSIMPs and MWCNTs could significantly enhance the toughness and tensile properties of the epoxy resin. Also, it was observed that the dominant factors for improving the fracture toughness of the ternary composites are crack deflection/arresting as well as enlarged plastic deformation around the growing crack tip induced by the combination of rigid and soft particles. The Response Surface Methodology (RSM) with central composite design (CCD) was utilized to study the effects of the amounts of CSIMPs and MWCNTs on the physicomechanical properties of the epoxy resin. The proposed quadratic models were in accordance with the experimental results with correlation coefficient more than 98%. The optimum condition for maximum toughness, elastic modulus, and tensile strength was 3 wt% MWCNT and 1.03 wt% CSIMPs. The sample fabricated in the optimal condition indicated toughness, elastic modulus, and tensile strength equal to 2.2 MPa m^1/2^, 3014.5 MPa, and 40.6 MPa, respectively.

## Introduction

Epoxy resins, due to their great physicomechanical attributes such as good chemical resistance, outstanding adhesion, and exceptional dimensional stability, are of paramount importance in various structural applications^1^. However, due to their high density of three-dimensional molecular structure, hardly can they resist toward growth of crack. This serious drawback limits their applications in many critical fields where a notable fracture strength or low-temperature toughness is required, such as aerospace and automotive industries^2,3^. To deplete the severity of this issue, a plethora of different ingredients such as liquid rubbers, core–shell impact modifier particles (CSIMPs), and rigid organic and inorganic ingredients were used as impact modifiers particles (IMPs) of epoxy resin^4–7^.

Generally speaking, soft IMPs (i.e. liquid rubbers and CSIMPs) could endanger significant improvement in the toughness of epoxy resin through various mechanisms such as deboning, cavitation, and crazing^6,8^. Nonetheless, incorporation of such IMPs in epoxy formulation could result in deterioration of other desired properties of the host polymer (such as modulus, stiffness, and tensile strength)^9^. On the other hand, rigid IMPs not only could enhance the toughness of epoxy resin by crack pinning, crack deflection, and deflection/bifurcation effect of growing crack, but also could boost stiffness and modulus of the host polymer^10^. However, to reach a satisfying level of toughness, it is inevitable to use a relatively high dosage of such IMPs in the epoxy formulation. This obligation causes a dramatic increase in viscosity and subsequently inroads serious difficulties in the processability of the obtained composite^11^.

Regarding the mentioned limitations of IMPs and their inherent differences from the viewpoint of toughening mechanisms, in the last decade, hybridization of different rigid and soft IMPs embedded into epoxy polymer matrix provided outstanding opportunities to the researchers for developing high-performance nanocomposites with enhanced toughness and physicomechanical properties^11–16^. The obtained ternary nanocomposite could overcome the mentioned drawbacks and cause synergistic influences on the toughness and other physicomechanical properties of the obtained composite products.

It is no doubt that outstanding attributes of carbon nanotubes such as their high tensile strength and stiffness, could remarkably enhance fracture toughness and physicomechanical properties of polymers^17^. Thus, among different rigid particles, it has been demonstrated that using carbon nanotubes as rigid fillers along with other soft IMPs could culminate a notable enhancement in fracture toughness of the obtained composites. Río et al. showed that the addition of a mixture of rigid carbon nanotube and soft polystyrene-polybutadiene-poly (methyl methacrylate) triblock copolymers led to the dramatic increase in both fracture toughness and fracture energy of the epoxy resin^18^. In another attempt, it was revealed that using block copolymer could ameliorate the dispersion state of carbon nanotubes and causes a synergistic effect on the toughness of epoxy resin^19^. Jojibabu et al. showed that ternary composite of triblock copolymer, modified carbon nanotubes, and epoxy resin possess a remarkable improved toughness in comparison with binary composites^20^.

The technique of experimental design, due to its valuable assistance for comprehensive understanding of the process and interactive relationships between different variables, is known as a distinguished method in the engineering fields^21^. Among different methods, the response surface method, which is a collection of mathematical and statistical techniques, has been used extensively to study and optimize the effect of diverse determining factors on the various parameters^22^. To the best of current authors' knowledge, there is no single published numerical and systematic study about the synergistic effects of MWCNTs and CSIMPs on the toughness and tensile properties of epoxy resin. Thus, a systematic study of the influence of multiwalled carbon nanotubes (MWCNTs) and CSIMPa on the mentioned attributes of epoxy resin is the main objective of this work. For this purpose, response surface methodology was used to find an applicable approximation function for predicting and optimization of the toughness and tensile properties of composites with various amounts of CSIMPs and MWCNTs.

## Experimental

### Materials

Monomers, glycidyl methacrylate (GMA), methyl methacrylate (MMA), allyl methacrylate (ALMA) from Merck Chemical Co., and butyl acrylate (BA) from Fluka were purchased and used as received. Emulsogen APS 100 from Clariant, ter-butyl perbenzoate (TBPB) from Merck Chemical Co., sodium formaldehyde sulfoxylate (SFS), ethylene diamine tertaacetic acid (EDTA), iron (II) sulfate heptahydrate (FeSO_4_. 7H_2_O), and ammonium persulphate (APS) from Aldrich, were used without any further purification. MWCNTs with the purity of > 95%, length of 5–15 µm, and outer diameter of 10–20 nm was purchased from Nutrino (Tehran, Iran). The epoxy resin was standard diglycidylether of bisphenol A (DGEBA) (Epon828 from HEXION) with an epoxy equivalent molecular weight between 185 g/eq and 192 g/eq. The curing agent was cycloaliphatic polyamine hardener (Epicure F205 from HEXION) with hydroxyl equivalent weight of 102–106 g/eq. Deionized water (DIW) was used in all recipes.

### Characterizations

FT-IR spectrum was recorded on FT-IR BRUKER-IFS 48 spectrophotometer (Germany) using KBr pellet. Glass transition temperature (T_g_) of the core and shell portions of the prepared CSIMPs were determined according to ASTM D 3418-08, by differential scanning calorimeter, DSC-Netzsch (England). The prepared dried sample was scanned in the temperature range between − 100 and 140 °C at a heating rate of 10 °C/min under nitrogen atmosphere. The T_g_ was defined as the midpoint of deflection of the baseline from the DSC thermogram. Size and external morphology of the prepared latex particles were investigated by scanning electron microscopy (SEM) with MIRA3 instrument from Tescan (The Czech Republic). Also, SEM images of the fracture surfaces of the epoxy specimens were used to assess the toughening mechanisms. The internal morphology of the prepared latex particles was studied by transmission electron microscopy (TEM) with CEM 902A ZEISS with an accelerating voltage of 80 keV (Oberkochen, Germany). Tensile properties of the dog-bone-shaped specimens were obtained by a universal testing machine according to ASTM D-638. For each sample, the data reported are the average of five measurements. Fracture energy (G_IC_) and fracture toughness (K_IC_) were determined using a single-edge notch bend (SENB) specimen with dimensions of 6 × 12 × 52.8 mm. The tests were performed at a rate of 10 mm/min.

### Synthesis of the impact modifier particles

Soft impact modifier particles with core–shell morphology were prepared through emulsion polymerization technique in two stages. At the first step, elastomeric cores of poly (BA-ALMA) were prepared through batchwise emulsion polymerization. For this purpose, 160 g DIW, 1 g Emulsogen APS 100, 30 g BA, and 0.16 g ALMA were premixed and charged into a 250 mL, four-necked round bottom glass reactor equipped with reflux condenser, thermocouple, crescent Teflon-steel mechanical stirrer, and feeding inlets. The content of the reactor was deoxygenated by purging with nitrogen for 15 min. The temperature was raised to 80 °C and the polymerization reaction was started by the addition of solution of the initiator (0.04 g APS dissolved at 10 mL water). The conversion of the polymerization reaction was followed by gravimetric analysis of samples withdrawn from the reactor at regular time intervals. After 120 min, the final conversion of the polymerization reaction was above 99%.

In the second stage, copolymerization of MMA and GMA was occurred on the surface of the prepared elastomeric cores. For that, 200 g of the prepared latex, 9 g MMA, 1 g GMA, and 0.1 g TBPB charged into the reactor. The content of the reactor was mixed for 30 min at room temperature. Then, 10 mL of the aqueous solution of 0.02 g EDTA, 0.01 g FeSO_4_·7H_2_O, and 0.1 g SFS were added into the reactor. The redox emulsion copolymerization was proceeded for 3 h at 30 °C to reach conversion of > 98%. Then, the prepared latex was filtered through a 53 mm sieve and the filtrate was freezing for 12 h and freeze-drying for another 12 h.

### Experimental design and fabrication of toughened specimens

The CCD of RSM was used to assess the effects of the contents of MWCNTs and CSIMPs as independent factors on the fracture toughness and tensile properties of epoxy composites. Table 1 shows the ranges and levels of these factors. Total number of 13 experiments was designed by CCD algorithm which consists of 4 cube points, 4 axial points and 5 replications of the central points in cube to check the reproducibility variance.

**Table 1:** Real and coded levels of variables.

Independent factor	Symbol	Coded level				
		− 1.5	− 1	0	1	1.5
MWNT (wt%)	X_1_	0	0.5	1.5	2.5	3
CSIMP (wt%)	X_2_	0	0.5	1.5	2.5	3

Table 2 illustrates the details of the designed experimental conditions proposed by CCD. To find the best fitted model, the step-wise model fitting by Minitab 14 software was used. Equation 1, which is a second-order empirical quadratic polynomial model, shows the behavior of the system

**Table 2:** Experimental design matrix and responses.

Run	Factors		Response: Young’s modulus, E (MPa)		Response: tensile strength, S (MPa)		Response: fracture toughness, K_IC_ (MPa. m^1/2^)	
	wt% MWCNT	wt% CSIMP	Exp	Fit	Exp	Fit	Exp	Fit
1	0.5	0.5	1480.680	1556.91	26.02	26.17	0.89	0.78
2	1.5	1.5	1934.51	1882.85	34.45	34.36	2.27	2.18
3	1.5	1.5	1863.62	1882.85	34.96	34.36	2.07	2.18
4	0.5	2.5	1523.7	1612.02	31.46	31.32	2.24	2.35
5	1.5	1.5	1892.91	1882.85	33.39	34.36	2.13	2.18
6	2.5	0.5	2917.6	2958.44	40.35	40.73	1.83	1.78
7	3	1.5	2745.4	2711.6	38.13	37.86	2.61	2.55
8	1.5	0	2438.14	2388.79	34.02	33.73	0.98	1.11
9	1.5	3	1762.94	1697.49	30.8	30.88	3.61	3.44
10	2.5	2.5	1928.68	1981.59	31.7	31.8	3.16	3.32
11	1.5	1.5	1927.73	1882.86	34.08	34.36	2.29	2.18
12	0	1.5	1464.27	1383.27	26.55	26.6	1.07	1.08
13	1.5	1.5	1824.21	1882.85	34.99	34.36	2.17	2.18

1 $$Y={b}_{0}+\sum_{i=1}^{k}{b}_{i}{x}_{i}+\sum_{i=1}^{k}{b}_{ii}{x}_{i}^{2}+\sum_{i}^{k}\sum_{j}^{k}{b}_{ij}{x}_{i}{x}_{j},$$

where Y is the predicted physicomechanical response, b_o_ is the constant, b_i_ is the linear effect of the factor x_i_ (i = 1, 2 and 3), b_ii_ is the quadratic effect of the factor x_i_ and b_ij_ is the linear interaction effect between the input factors, x_i_ and x_j_.

To evaluate the effect of CSIMPs and MWCNTs on the physicomechanical properties of epoxy resin, specimens with different contents of these reinforcing fillers were fabricated according to the Table 2. For this purpose, definite amounts of MWCNTs and CSIMPs were added to 100 g of epoxy resin and stirred to form a uniform mixture. Then, to ensure that the fillers were uniformly distributed in the epoxy matrix, the mixture was processed by a three-roll milling (EXAKT 80E, Exakt Technologies, Germany) for 20 min via a three-roll grinder. The three-roll milling was used in gap mode with a 5 mm distance configuration between the center and apron roller. The speed of the apron roller was 260 rpm and the roller speed ratio was 6:2:1. Then, 58 g Epicure F205 as a curing agent was added to the mixture and stirred with a glass rod. Finally, the prepared mixtures were degassed by a centrifugal deaerator for 10 min at 3000 rpm. The mixtures were poured into silicon molds and a two-step curing procedure was carried out; 2 h at 40 °C then 4 h at 120 °C using a ramp rate of 10 °C/min.

## Results and discussion

### Preparation of impact modifier particles with core–shell morphology

**Figure 1:**
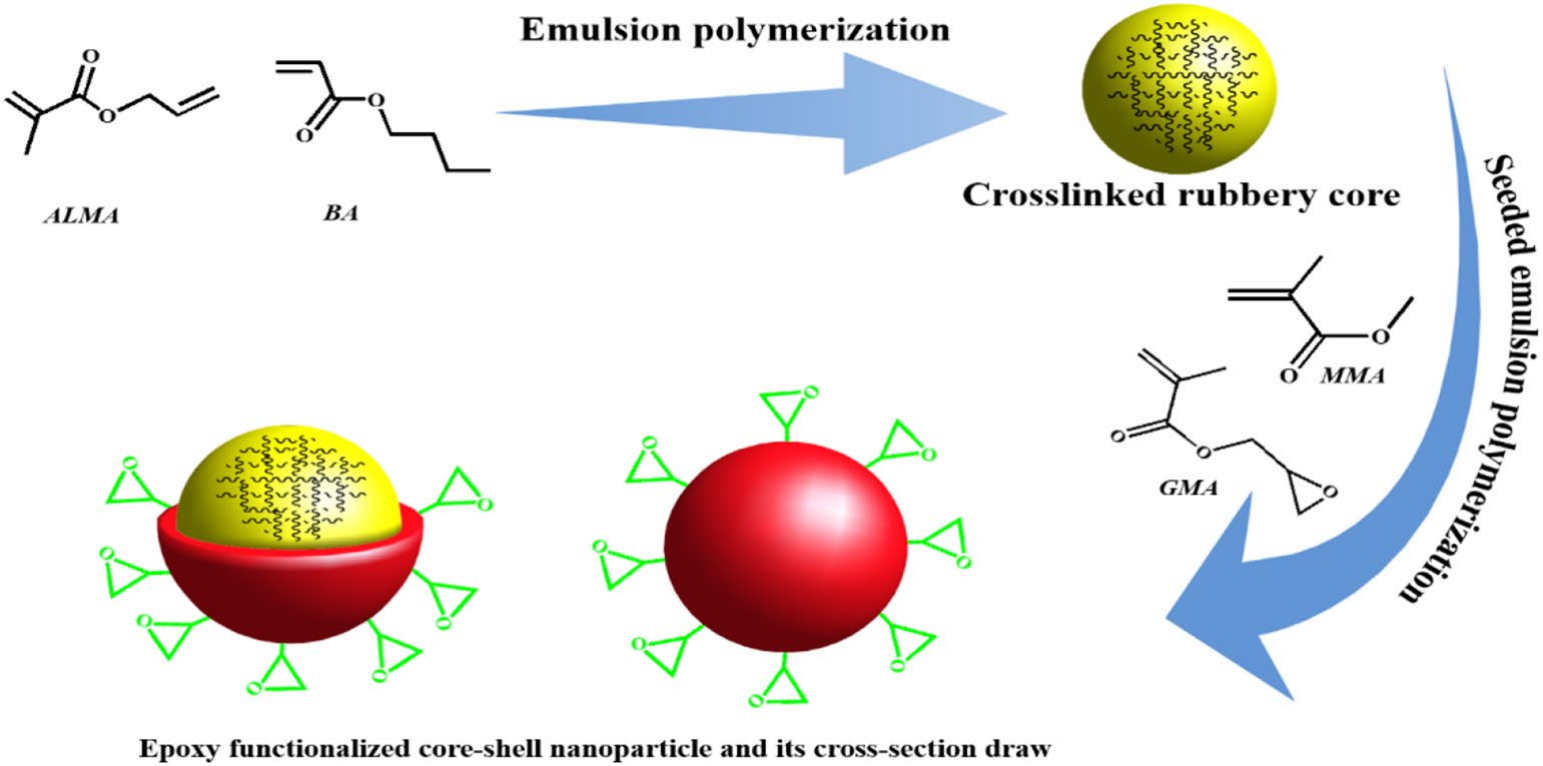
Schematic illustration of preparation steps of CSIMPs.

The capability of emulsion polymerization in preparing colloidal particles with disparate morphologies, physical, and chemical attributes makes it a unique and enticing polymerization method^23^. Here, thanks to these features, impact modifier particles with elastomeric core and epoxy-functionalized plastic shell were prepared successfully. A schematic illustration of the preparation steps of the CSIMPs is shown in Fig. 1.

It is well known that using monomers such as ALMA, which has unsaturated bonds with different reactivity, could significantly enhance grafting efficiency, gel content, and structural stability of CSIMPs^24^. Linked to these rationales, in this work, ALMA was chosen as a graflinking monomer in the formulation of the prepared elastomeric cores of CSIMPs. To fabricate the plastic shell, redox emulsion copolymerization of MMA and GMA was occurred at the interfacial region of elastomeric cores and aqueous medium. It was suggested that using a swelling time for dissolving monomers in poly (BA-ALMA) colloidal particles, as well as proceeding the radical formation reactions at the interfacial region, could effectively minimize the probability of micellar and homogeneous nucleation^25^. Also, it is surmised that such swelling time could significantly enhance the grafting reaction between the elastomeric core and the glassy shell. This reaction could enhance the stability of the formed shell toward dissolution and separation from the elastomeric core during mixing of CSIMPs with epoxy polymer.

**Figure 2:**
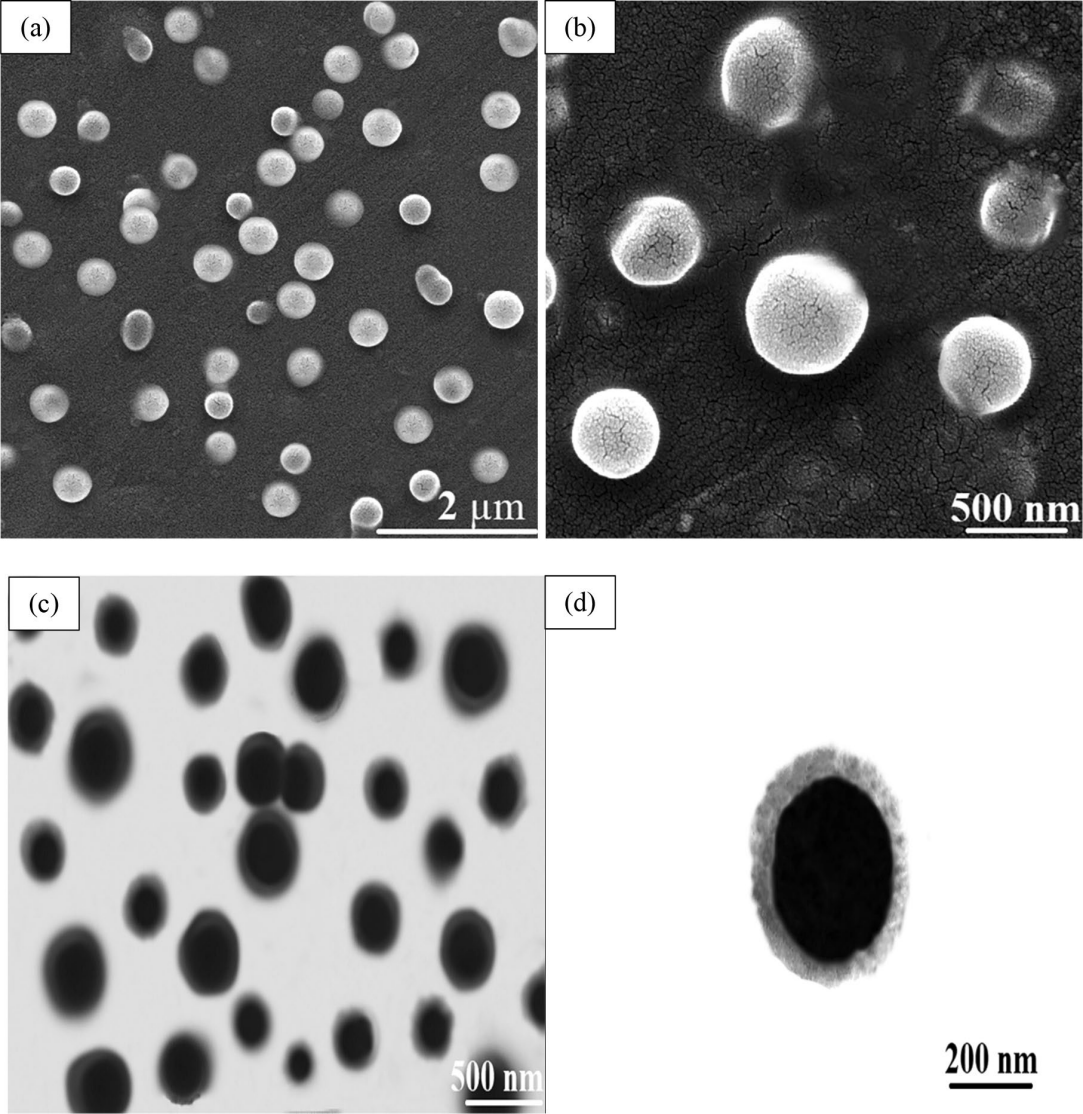
SEM (**a**,**b**) and TEM (**c**,**d**) images of the prepared IMPs.

The electron microscopy results of the prepared CSIMPs (Fig. 2) manifest that the employed synthesis method could successfully fabricate CSIMPs with an average particle size of 337 nm. Also, TEM images revealed that the average sizes of the core and shell are 246 nm and 45 nm, respectively. There are a plethora of reports about significant influences of the diameter of the elastomeric core and the thickness of the glassy shell on the performance of CSIMPs^26–29^. For example, it has been demonstrated that by decreasing the particle size of various elastomeric impact modifiers such as carboxyl-terminated butadiene acrylonitrile (CTBN) or CSIMPs their resistance towards cavitation increased^26^. Kim et al. showed that due to the difficulty in cavitation of rubbery core, toughening effectiveness of poly (butyl acrylate) core- poly (methyl methacrylate) shell particles decreased for particle sizes smaller than 0.2 µm^27^. However, fracture toughness was constant in the bigger particle sizes ranges. Here, the measured average particle size of the prepared poly (ALMA-BA) core is in good accord with the size of the elastomeric core of the other prepared CSIMPs which could remarkably enhance the fracture toughness of epoxy polymer^9,27–29^. Thus, it is surmised that the fabricated elastomeric particles could efficiently ameliorate the toughness of epoxy resin through toughening mechanisms such as induced plastic deformation and cavitation.

The most prominent role of the glassy shell of CSIMPs is making a compatible layer between the elastomeric core and epoxy matrix. This layer provides a stress bridge for transferring of the energy of the imposed load from the matrix to the rubbery core of CSIMPs^29^. Thus, the lower thickness of the shell could remarkably facilitate the transferring stress from polymer matrix toward the rubbery core and enhance the performance of the CSIMPs. On the other hand, the adequate thickness of the formed glassy shell could guarantee the compatibility between CSIMPs and epoxy matrix and enhance the dispersion state of the CSIMPs by preventing their coalescence during the drying and compounding process. The low measured average thickness of the shell and the free-flowing state of the synthesized CSIMPs at their powdery form substantiated that the thickness of the shell is in a suitable range for meeting the cited challenges^30^.

**Figure 3:**
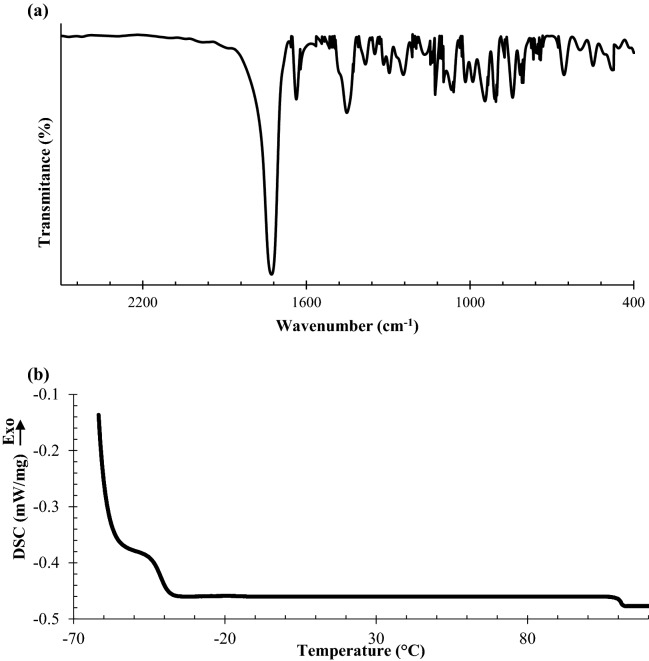
(**a**) FTIR spectrum and (**b**) DSC thermogram of the prepared CSIMP.

Figure 3a represents the FTIR spectra of the prepared CSIMPs. The adsorption peaks at 1730 cm^−1^ and 1160 cm^−1^ are attributed to the C=O and C–O stretching vibrations of ester groups, respectively. The absorption peaks at 1230–1280 cm^−1^, 815–950 cm^−1^, and 750–770 cm^−1^, correspond to the symmetric stretching vibration and bending mode of the epoxy groups and confirm that the exploited GMA monomer could successfully functionalize the CSIMPs by epoxy rings^20^.

DSC thermogram of the prepared CSIMPs revealed two main transition temperatures (Fig. 3b). The first transition (≈ − 41 °C) was related to the T_g_ of the elastomeric core. The observed difference between the measured T_g_ of the elastomeric core and reported values for poly (butyl acrylate) could be due to its crosslinked structure and induced restriction by the grafting glassy shell of CSIMPs^25^. The second glass transition temperature (≈ 109 °C) corresponds to the grafted poly (MMA-GMA) shell. The observed distinct differences between measured T_g_s of the core and shell portions of CSIMPs confirmed that exploited monomers in each step of the preparation procedure could form discrete phases^31^.

### Modeling and optimization using response surface methodology

The results in Table 2 were used to fit the polynomial models representing the Young’s modulus (E), tensile strength (S) and fracture toughness (K_IC_) of the toughened specimens (responses) as a function of the contents of MWCNTs (wt%; X_1_) and CSIMPs (wt%; X_2_). The following equations express the overall predictive model in terms of the coded variables:

2 $$ {\text{Y}}_{{{\text{Young's modulus}}}} = \, 1882.9 \, + \, 664.2{\text{ X}}_{1} - 345.7{\text{ X}}_{2} + \, 164.6{\text{ X}}_{1}^{2} + 160.3{\text{ X}}_{2}^{2} - 580.5{\text{ X}}_{1} {\text{X}}_{2} , $$

3 $$ {\text{Y}}_{{{\text{Tensile strength }}}} = { 34}.{364 } + { 5}.{\text{633 X}}_{{1}} - { 1}.{\text{423 X}}_{{2}} - { 2}.{\text{132 X}}_{{1}}^{{2}} - {2}.0{\text{59 X}}_{{2}}^{{2}} - { 7}.{\text{935 X}}_{{1}} {\text{X}}_{{2}} , $$

4 $$ {\text{Y}}_{{{\text{Fracture toughness}}}} = { 2}.{182 } + \, 0.{\text{73826 X}}_{{1}} + { 1}.{\text{16762 X}}_{{2}} - \, 0.{\text{36754 X}}_{{1}}^{{2}} + \, 0.0{\text{9146 X}}_{{2}}^{{2}} - 0.0{\text{1181 X}}_{{1}} {\text{X}}_{{2}} . $$

The importance and statistical significance of the quadratic models were evaluated by the analysis of variance (ANOVA), which has been presented in Table 3. The ANOVA includes some statistic factors such as lack of fit, R^2^, and adjusted R^2^. The ANOVA results (Table 2) indicated that the models were highly significant, as P-value for the models was 0.000. The high R^2^ values of the models demonstrated that they were capable of accurately predicting the mechanical properties of the prepared epoxy samples in the studied range. The insignificance “lack of fit” with P-value of higher than 0.05 indicated that in the models, LOF is meaningless and the models have appropriate accuracy for predicting the results. All terms in the regression models are not equally important. The significance of each coefficient was determined by *P* values (probability), which are listed in Table 3. The probability values less than 0.05 call for the rejection of the null hypothesis, indicating that the particular term significantly affects the mechanical properties of the coatings.

**Table 3:** Analysis of variance for the models and estimated regression coefficients.

Term	Coef	P-value
**(a) Response: Young’s modulus (E)**		
Constant	1882.9	0.000
wt% MWCNT	664.2	0.000
wt% CSIMP	− 345.7	0.000
wt% MWCNT × wt% MWCNT	164.6	0.029
wt% CSIMP × wt% CSIMP	160.3	0.032
wt% MWCNT × wt% CSIMP	− 580.5	0.000
**(b) Response: Tensile strength (S)**		
Constant	34.364	0.000
wt% MWCNT	5.633	0.000
wt% CSIMP	− 1.423	0.002
wt% MWCNT × wt% MWCNT	− 2.132	0.002
wt% CSIMP × wt% CSIMP	− 2.059	0.002
wt% MWCNT × wt% CSIMP	− 7.935	0.000
**(c) Response: fracture toughness (K**_**IC**_**)**		
Constant	2.18200	0.000
wt% MWCNT	0.73826	0.000
wt% CSIMP	1.16762	0.000
wt% MWCNT × wt% MWCNT	− 0.36754	0.000
wt% CSIMP × wt% CSIMP	0.09146	0.425
wt% MWCNT × wt% CSIMP	− 0.01181	0.942

(a) R-Sq = 98.4%, R-Sq(adj) = 97.2%, P-value of Lack-of-Fit = 0.073, P-value of Regression = 0.000.

R-Sq = 98.9%, R-Sq(adj) = 98.1%, P-value of Lack-of-Fit = 0.846, P-value of Regression = 0.000.

(c) R-Sq = 98.2%, R-Sq(adj) = 97%, P-value of Lack-of-Fit = 0.113, P-value of Regression = 0.000.

**Figure 4:**
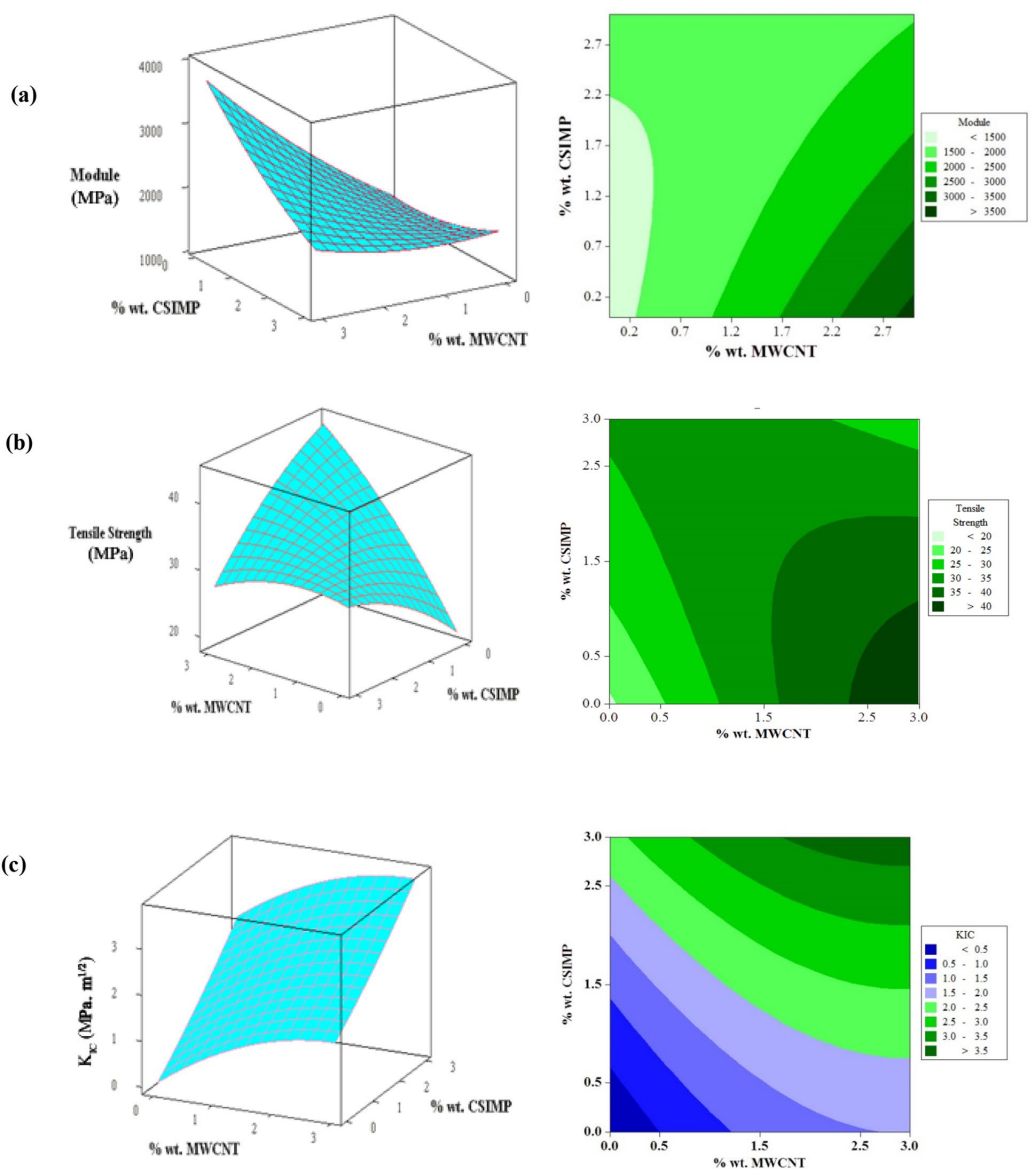
Response surface and Contour plots of tensile properties and toughness of epoxy composites.

The obtained response surface and contour plots provide a procedure to predict the mechanical properties of the prepared epoxy specimens reinforced with various amounts of MWCNTs and CSIMPs. As it can be seen obviously in Fig. 4a, by increasing the content of MWCNTs, due to their intrinsic rigidity and significantly high Young’s modulus, the E values of the fabricated epoxy composites increased remarkably^32^. Also, it can be seen that the addition of CSIMPs could not effectively alter Young’s modulus of the host polymer up to 2.2 wt%. The plausible explanation for this observation could be due to the relatively low dosage of the used CSIMPs and also their ability to establish covalent linkages with epoxy matrix^33^. The latter phenomena could effectively form spatial hindrance for polymer chains and increase the crosslink density of the final composite^34^. Nevertheless, this effect could partially be compensated by the elastomeric core of the CSIMPs and result in the observed plateau region of modulus as a function of the content of CSIMPs. However, at higher levels of CSIMPs (> 2.2 wt%), an increase in the modulus of the prepared composites was observed which could be attributed to the increasing in the content of the glassy functional shell of the CSIMPs in the composites structures^35^. As it can be seen clearly from the obtained contour plot, the measured modulus of the fabricated ternary composites could be explained by the cited rationales for individual effects of MWCNTs and CSIMPs.

It is well known that introducing MWCNTs in the epoxy matrix could exert significant influence over the tensile strength of the obtained composite^36^. This influence could be seen as a remarkable improvement in the tensile strength by increasing the content of MWCNTs in Fig. 4b. Also, it is seen that the addition of CSIMPs causes an improvement in the tensile strength of the host polymer. This observation could be related to two pivotal traits of CSIMPs; (i) the ability of the plastic shell for the creation of covalent bonds with epoxy matrix, (ii) crosslinked attribute of the core, which could guarantee the structural stability of CSIMPs^28^. The obtained S values of ternary composites revealed that the effect of CSIMPs on the obtained results is more prominent than MWCNTs. In other words, at a constant concentration of MWCNTs, the addition of CSIMPs render a significant decrease in the tensile strength of the obtained nanocomposite, which could be attributed to the elastomeric portion of CSIMPs.

The influence of the contents of MWCNTs and CSIMPs on the fracture toughness of epoxy resin is shown in Fig. 4c. It can be seen clearly that both of the added ingredients could significantly increase the K_IC_ of epoxy resin. However, as it is expected, the influence of CSIMPs is more prominent than MWCNTs. Moreover, it is observed that for the binary composites of MWCNTs/epoxy the measured fracture toughness is not directly proportional to the amounts of MWCNTs. This observation is probably due to the dominant aggregation of MWCNTs at higher concentration of this material^37^. To put it in another way, it is well-known that due to the enormous specific surface area of MWCNTs, they have strong tendency for creating clusters within the epoxy matrix. The formed clusters of MWCNTs could decrease the interfacial surface area between the epoxy resin and rigid fillers, which consequently can reduce their interactions with growing cracks and decrease the toughening performance of MWCNTs. An enticing additional insight is the synergistic toughening effects of MWCNTs and CSIMPs. It can be seen conspicuously that the ternary composite with 3 wt% MWCNTs and 3 wt% CSIMPs possesses the highest value of fracture toughness. This observation shows that the employed rigid and soft fillers could exert significant positive influence over the fracture toughness of epoxy resin. It is substantiated that the dispersion state of MWCNTs could be improved significantly by the addition of polymeric impact modifiers^18^. Here, it is suggested that the added CSIMPs could decrease the aggregated structures of MWCNTs and facilitate their dispersion in the epoxy matrix.

Optimization of effectual factors was mainly done to define the optimum formulation that caused the achievement of maximum E, S, and K_IC_. The optimum values of selected factors were obtained by solving the regression equations (Eqs. 2–4). The optimum formulation for achieving the cited aims was found to be as follows: MWCNTs (wt%) = 3 and CSIMPs (wt%) = 1.03. The maximum mechanical properties predicted by using the optimum values of factors were E = 3014 MPa; S = 40.57 MPa, and K_IC_ = 2.2 MPa m^1/2^. The tensile properties and toughness of the ternary composite in optimum condition obtained through the experiment were E = 2970 MPa; S = 41.2 MPa, and K_IC_ = 2.1 MPa m^1/2^, which were in good accordance with the expected results. It can be concluded that the response surface methodology is a trustworthy tool for optimizing the mechanical properties of epoxy coatings.

### Fracture surface

**Figure 5:**
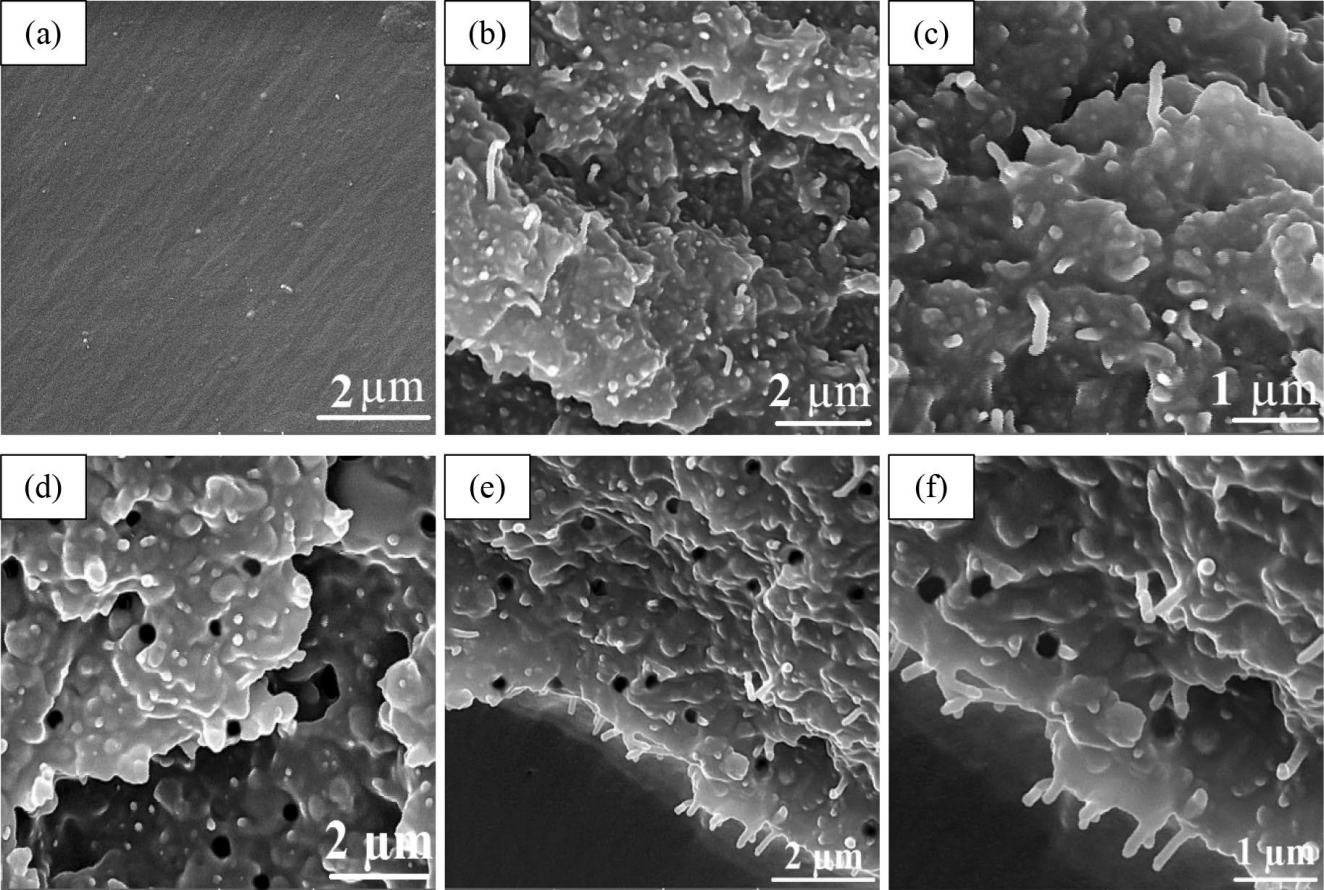
Fractographs of (**a**) neat epoxy, (**b**,**c**) epoxy composite with 1 wt% MWCNTs, (**d**) epoxy composite with 03% CSIMP, (**e**,**f**) composite with 0.5% MWCNT and 1.5% CSIMP.

To assess the effect of MWCNTs and CSIMPs on the fracture toughness of the ternary composite, the fracture surface of the epoxy polymer, binary, and the optimized ternary composites were comparatively examined by SEM. As it can be seen in Fig. 5a, the neat epoxy sample exhibits a typical fracture surface for unfilled epoxy resins, i.e., smooth fracture surface with the fine line patterns which indicates generally uninterrupted crack propagation and low fracture toughness^37^. On the other hand, for binary epoxy composite with 1.5 wt% MWCNTs (Fig. 5b), a rough fracture surface indicating a ductile fracture of the composite is observed. It has been reported that the addition of MWCNTs to epoxy matrix could significantly increase the fracture toughness of host polymer by introducing restrictions to crack propagation and increasing the amount of plastic deformation^38^. In other words, the propagating crack tips could not break the strong MWCNTs, and consequently, they force to arrest or change their directions. Also, the high magnification SEM fractograph of this specimen (Fig. 5c) shows a plethora of pulled-out nanotubes with curved pattern which indicate a great deal of difficulty in crack initiation and propagation within the matrix of MWCNT containing samples in comparison with bared epoxy matrix^39^. Therefore, through the process of pulling out of MWCNTs from the epoxy matrix, a great deal of dissipation of energy has occurred which caused a remarkable amelioration in fracture toughness of the prepared nanocomposites.

It is well known that the dispersion state of MWCNTs could significantly affect the toughness and other physicomechanical properties of the epoxy polymer. Thus, it is expected that the observed agglomerations of MWCNTs in the fractograph of the binary composite (Fig. 5b) could act as weak and stress concentration areas and deteriorate physicomechanical properties of the epoxy polymer. By considering that such aggregated structures become more prevailing at the higher concentration of MWCNTs, it is plausible that the devastating effects of such conglomerations are more prominent at higher amounts of MWCNTs. Such phenomenon was observed as the deteriorative influence of MWCNTs on the S and K_IC_ of the epoxy composites at the Response surface and Contour plots in Fig. 4.

Figure 5d shows the fractograph of the binary composite of epoxy/CSIMPs. Rough fracture surface as well as spherical holes, which are related to the deboning of CSIMPs and subsequent plastic deformation of matrix indicate a ductile fracture of the prepared specimen^40^. It is surmised that stress-activated shear yielding in the high-stress region around CSIMPs leads to the deformation of their rubbery portion and cavitation phenomenon, which in turn could significantly boost the toughness of epoxy resin. In addition, as it can be seen obviously, the sizes of the holes are considerably bigger than the measured size of CSIMPs in SEM images (Fig. 2). This observation is probably due to the massive plastic deformation of the matrix around the AIMPs^41^.

Fractographs of toughened samples with MWCNTs and CSIMPs conspicuously show plastic deformation, crack deflection and pull-out MWCNTs (Fig. 5e,f). This observation surmised that a combination of crack deflection and inhibition of MWCNTs along with cavitation of CSIMPs could have synergistic toughening effects on the epoxy matrix and enhance its toughness remarkably. Also, it is observed that the conglomerations of MWCNTs at the ternary composite are considerably lower than MWCNTs/epoxy binary composite. As explained in Sect. 3.2, the probable explanation for such phenomenon could be due to the positive effect of CSIMPs on the dispersion state of MWCNTs.

## Conclusion

In this study, for the first time CCD/RSM design was adopted to determine the optimum condition for tensile properties and fracture toughness of epoxy/MWCNT/CSIMPs ternary composite. The CCD/RSM prepared sufficient statistical data to fit a quadratic model in terms of the wt% MWCNTs and wt% CSIMPs in epoxy resin, on the tensile and toughness properties. The significance of the proposed models was indicated by the p-value of Regression < 0.0001 and P-value of LOF > 0.05. Analysis of variance showed good coefficient of determination values, R^2^, (> 98) for the studied properties. The optimum condition for maximum Young’s modulus, tensile strength, and toughness of the epoxy composite obtained from RSM was 3 wt% MWCNTs and 1.03 wt% CSIMPs in the epoxy matrix. The sample prepared in optimal conditions indicated module = 3014 MPa, tensile strength = 40.57 MPa, and K_IC_ = 2.2 MPa m^1/2^. The obtained results indicated that the optimized ternary composites possess a good balance between Young’s modulus, tensile strength, and toughness, which could not be achieved by other binary ones. The SEM images of the fractographes substantiated that combination of MWCNTs and CSIMPs could merge their toughening mechanisms and cause a synergistic toughening effect on the obtained ternary composite.
